# Early Determinants of Obesity: Genetic, Epigenetic, and In Utero Influences

**DOI:** 10.1155/2012/463850

**Published:** 2012-05-31

**Authors:** Kyung E. Rhee, Suzanne Phelan, Jeanne McCaffery

**Affiliations:** ^1^Department of Pediatrics, University of California, 4305 University Avenue, Suite 590, San Diego, CA 92105, USA; ^2^Department of Kinesiology, California Polytechnic State University, San Luis Obispo, CA 93407, USA; ^3^Department of Psychiatry and Human Behavior, Warren Alpert Medical School of Brown University, Providence, RI 02903, USA

## Abstract

There is an emerging body of work indicating that genes, epigenetics, and the in utero environment can impact whether or not a child is obese. While certain genes have been identified that increase one's risk for becoming obese, other factors such as excess gestational weight gain, gestational diabetes mellitus, and smoking can also influence this risk. Understanding these influences can help to inform which behaviors and exposures should be targeted if we are to decrease the prevalence of obesity. By helping parents and young children change certain behaviors and exposures during critical time periods, we may be able to alter or modify one's genetic predisposition. However, further research is needed to determine which efforts are effective at decreasing the incidence of obesity and to develop new methods of prevention. In this paper, we will discuss how genes, epigenetics, and in utero influences affect the development of obesity. We will then discuss current efforts to alter these influences and suggest future directions for this work.

## 1. Introduction

Childhood obesity is a worldwide public health concern with several etiologic influences. The intergenerational relationship between parent and child obesity has been welldescribed [1], and twin studies have estimated that genes are responsible for 40–75% of the phenotypic variance of obesity [2–4]. However, evolutionary changes in the genome cannot explain the tremendous increase in obesity prevalence over the past 30 years. Most likely, the genetic susceptibility to obesity has always existed, but is now becoming more evident due to the influence of the obesogenic environment. This gene-environment interaction likely drives the obesity epidemic and helps to explain individual variations in obesity development, that is, why one sedentary child eating a high-fat diet becomes obese and another child who eats similarly does not. In addition to this interaction, animal studies have suggested that epigenetic influences, like nutritional exposure in utero, can alter one's gene expression and further impact the risk for obesity. Understanding how certain factors can modify gene expression during fetal development and early childhood can help to explain the rapid rise of obesity during a time when minimal to no populationwide genomic changes have occurred. This knowledge may ultimately help us prevent the development of obesity in a significant number of children. In this paper, our goal is to outline the influence of genetic, epigenetic, and in utero factors on the development of childhood obesity. With this information, we can lay the groundwork for future obesity prevention efforts.

## 2. Genetics

Over the past several years, many discoveries have been made regarding the genetic variation that influences complex diseases like cardiovascular disease and obesity [5]. These new discoveries have largely resulted from genomewide association studies where the application of high-throughput genotyping of millions of genetic markers enables researchers to examine genetic associations on a genomewide basis. Recent reports indicate that at least 32 genes contribute to common forms of obesity [6–11]. A number of these have also been confirmed as contributors to pediatric obesity [10, 12]. Many of these genes are thought to be related to the development of obesity through the dysregulation of leptin or other metabolic hormones in the body. However, a majority of the newly discovered genes are expressed in the brain, emphasizing the role of the central nervous system in obesity risk [8].

The obesity-associated variant in the *FTO *gene has garnered particular interest in pediatrics because of its association with increased weight and ponderal index at 2 weeks of age [13]. FTO is located on the long arm of chromosome 16 and is expressed in the brain, specifically the hypothalamic nuclei. Those who are homozygous for the at-risk allele have been found to be up to 3 kg heavier than those who do not have the allele [10]. This weight gain is likely due to the gene's involvement in the regulation of energy intake. According to recent studies, individuals carrying the at-risk allele prefer energy dense food [14], have reduced feelings of satiety [15], display loss of control over eating [16], consume more fat and calories (even after adjusting for BMI) [17], and display a greater tendency towards consuming palatable foods after eating a meal [18]. Thus, *FTO* seems to predispose individuals to greater caloric intake and reduced feelings of satiety. On the other hand, *FTO* does not seem to be related to energy expenditure. A meta-analysis of 45 studies found that adults who were physically active could attenuate the odds of obesity associated with *FTO* by almost 30% [19]. Thus carrying a gene for obesity does not necessarily predestine one to become obese [20] but, rather, increases one's risk in the face of an obesogenic environment. Unfortunately, this attenuation of obesity risk via physical activity was not seen in children [19]. Nevertheless, taken together, these results suggest that interventions targeting food availability or physical activity may mitigate the increased genetic risk found in those with the *FTO *gene.

In addition to common forms of obesity, several rare, monogenic forms of obesity have been described including Prader-Willi syndrome and Bardet-Biedl syndrome. One of the most common forms of monogenic obesity, representing up to 5% of severe childhood obesity, is linked to melanocortin-4 receptor (*MC4R) *gene mutations [21, 22]. The *MC4R* gene is located on the long arm of chromosome 18 and encodes a G-protein-linked receptor [23] that is primarily expressed in the brain, more specifically in the paraventricular nucleus of the hypothalamus. The receptor is typically stimulated by insulin and leptin release which signals proopiomelanocortin (POMC) neurons to synthesize and release alpha-melanocyte stimulating hormone (*α*-MSH). When *α*-MSH binds to *MC4R*, individuals begin to feel satiated and increase energy expenditure through thermogenesis. Mutations in the *MC4R *gene can decrease this response and lead to rare familial forms of severe obesity [21, 24]. Mutations lead to excess intake of energy, and individuals with this mutation have displayed a preference for foods high in total and saturated fats [25]. Unlike *FTO* however, it is also associated with decreased energy expenditure [26]. Thus mutations in *MC4R* seem to impact both sides of the energy equation, that is, by increasing consumption and decreasing expenditure. While the gene has an autosomal dominant mode of transmission, it also has incomplete penetrance, thereby making clinical diagnosis difficult. Its incomplete penetrance however suggests that other factors, possibly epigenetic or environmental, may be impacting phenotypic expression. Given the different mechanism of action associated with this gene, it is important not to lose sight of the rare but often severe forms of monogenic obesity as we consider which environmental manipulations to pursue.

## 3. Epigenetics

 While several genes have been linked to excess weight gain, the existence of these genes cannot explain the rapidly growing prevalence of obesity. Epigenetics is the study of how early environmental influences affect gene expression and ultimately growth, development, and risk for disease without changing the underlying DNA sequence. Epigenetic mechanisms involve chemical processes such as DNA methylation, covalent modifications to histones that bind to DNA, and chromatin folding that can change gene expression and chromatin structure without changing the nucleotide sequence. Epigenetic changes can sometimes promote the expression of a gene that has typically been silent or silence a gene that is usually active. Examples of this phenomenon are widely evident as stem cells differentiate into specific somatic cells, like heart tissue or brain tissue, and genes are hypo- or hypermethylated to become cancerous.

Influences that may alter gene expression include inflammation, aging, oxidative stress, and hypoxia [27]. Scientists are also examining whether other environmental factors like maternal obesity and nutritional quality before or during pregnancy can cause epigenetic changes in the fetal genome and subsequently increase the risk of a child becoming overweight. Nutritional components that may influence the methylation of epigenetically susceptible loci include folic acid, vitamin B6 and 12, selenium, choline and betaine, methionine, soy genistein, bisphenol A, tocopherols, diallyl disulfide in garlic, and tea polyphenols [28].

## 4. Animal Studies

For many years, scientists have been using animal models to manipulate the genome and demonstrate how environmental exposures can alter the expression of genes so that certain characteristics, such as risk for obesity, can be suppressed or expressed. The agouti mouse, which was genetically altered to have yellow fur, has been used in many of these studies. For example, scientists have demonstrated that supplementing the mouse diet with methyl donors (like dietary folates, vitamin B_12_, methionine, and choline) can alter the expression of their genome and revert their coat color back to black [29]. While these epigenetic changes may or may not be passed down to the next generation, it does appear that it can be re-established in subsequent generations with continued dietary supplementation [30].

Interestingly, the yellow agouti mouse is also heavier and has a higher risk for hyperinsulinemia, diabetes, and tumors. The tendency for offspring of the yellow agouti mouse to be overweight is exacerbated if dams (mothers) are overweight. Supplementing the dam's diet with methyl donors, which has previously been shown to revert the offspring's coat color back to black, also prevented the future transmission of obesity to the offspring [31]. Thus changes in diet may be epigentically altering their genome so that these mice are phenotypically black and lean again. In addition to folate and vitamin B_12_, genistein, the major phytoestrogen in soy, has been shown to reduce the risk of obesity in the agouti mouse [32]. Whether or not genistein has similar effects on fat mass in humans is unknown. However, it is postulated that its methylating activity may explain the difference in cancer rates between Asians and Westerners. While studies that supplement human diets with methyl donors would be difficult to conduct, these animal studies suggest that the diet of pregnant mothers may be altering gene expression via epigenetic mechanisms and would therefore play an important role in influencing the health of future offspring.

The energy density of the food consumed during gestation may also be an important factor in determining one's phenotypic expression. Offspring of rats that are genetically susceptible to diet-induced obesity (DIO) are known to be heavier than offspring of rats who are diet resistant (DR). DIO dams who consume a high-energy diet or chow (normal energy diet) during gestation generally have offspring who are heavier than those from DR mothers exposed to either diet [33]. However, the exposure to high calorie diets during the gestational period exacerbated the risk of obesity in the DIO offspring such that those who were exposed to high-energy diets had the highest birth weight (compared to DIO offspring of dams on chow diets and DR offspring of dams on high calorie or chow diets) and demonstrated the greatest weight gain during the subsequent weeks despite being on similar diets as the DR offspring.

Another rat study, controlling for genetic influence, amount of gestational weight gain in the dams, and any post-natal effects from the mother via lactation, also found that the energy density of the dam's diet affected the offspring's weight trajectory, particularly if the offspring was exposed to a high-energy diet [34]. When examining the gene expression of adipose tissue and hypothalamus, it appeared that exposure to high-energy diets in utero caused an upregulation of certain gene products in the offspring which resulted in more efficient storage of the excess energy they consumed postnatally [35]. Other studies similarly suggest that maternal food intake that leads to excess gestational weight gain and associated metabolic changes in the in utero environment are affecting hypothalamic expression of genes involved in energy regulation [36, 37]. These studies demonstrate that an obesogenic in utero environment not only affects weight status at birth, but can increase the risk for obesity by priming the offspring's body to respond in a certain way to subsequent environmental or nutritional exposures. These results suggest that efforts to reduce one's exposure to high calorie diets, in utero and after the child is born, may be quite important to reduce one's risk for obesity.

Animal studies have also examined the influence of environmental exposures like nicotine and alcohol on offspring development. Findings suggest that nicotine exposure in utero increases postnatal weight gain [38] and may affect the development of hypothalamic function and subsequent appetite control through the upregulation of certain gene products [39, 40]. When exposed to alcohol in utero, rats appear to have increased gluconeogenesis and insulin resistance, making them more at risk for Type 2 diabetes [41–43]. These studies highlight the importance of other environmental exposures in explaining the rise in obesity and its associated comorbidities.

It is important to note that epigenetic changes can also occur postnatally. Since some tissues/organs and regulatory mechanisms are not fully developed at birth, there is still time to alter the expression of these genes once the offspring are born. In animal studies, cross-fostering, or suckling offspring with nonbiological mothers, changes the nutritional exposure of the offspring and can impact the development of obesity. For example, when DR offspring (who have no genetic risk for obesity) were fostered by obese DIO dams, they gained more weight than their counterparts who were fostered by DR dams. They also developed higher levels of insulin and leptin and had higher oral glucose tolerance test results [44]. Conversely, when DIO offspring were fostered by DR dams, they gained less weight compared to those suckled by DIO dams. They also had lower basal glucose levels [44]. Cross-fostering not only affected the offspring's weight and hormone levels, but changed mRNA expression of neuropeptides, insulin, and leptin receptors in the hypothalamus. The authors postulate that elements in the breast milk (such as higher insulin levels and lower polyunsaturated and monounsaturated fatty acid content in the milk of obese DIO dams) may have played a role in influencing these outcomes.

Additional studies have examined the impact of consuming high carbohydrate (HC) milk formula on the development of rats [45]. In these studies, the nutritional quality of milk was altered from its usual fat-rich state to a high-carbohydrate state without altering the total calorie content. After exposure to this milk in the immediate postnatal period, these pups developed chronic hyperinsulinemia and adult-onset obesity. At a molecular level, these pups had greater expression of mRNA related to pancreatic islet cell function and peptides of the brain (Neuropeptide Y, Agouti-related peptide, POMC, and MC4R). They were also able to transfer these traits (or risk profile) to their offspring without any further manipulations to the offspring's diet. These studies demonstrate the power of postnatal factors in modifying one's genetic predisposition and may further explain how environmental exposures are affecting the increasing prevalence of obesity.

## 5. Human Studies

Replicating these studies in humans is particularly challenging because of the great variety of epigenetic mechanisms that can influence gene expression as well as a lack of understanding as to where in the human genome and when during development these changes need to occur. Furthermore, studies in humans are complicated by the myriad of interpersonal and environmental factors that influence growth and development throughout life. Finally, there are many ethical considerations when conducting randomized controlled trials that manipulate exposures in utero and alter epigenetic expression. Conducting tests to confirm that epigenetic changes were made can also be difficult in humans, thereby limiting our ability to define clear relationships between exposures and outcomes. Given these limitations, animal studies will often be needed to infer underlying mechanisms.

However, there are some human studies that suggest epigenetic changes are occurring. One such line of research is with bariatric surgery patients. In an earlier study, Marceau et al. [46] compared the results of pregnancies in a group of women (mean BMI = 47.1 ± 8.3) *before* they had biliopancreatic diversion surgery (a form of bariatric surgery) and in another group of women *after* they had surgery (mean BMI = 30.9 ± 6.4). Of the 166 infants born in the postsurgery group, only 7.7% had fetal macrosomia (excessive weight at birth) compared to 34.8% of the infants born to mothers in the presurgery group (*n* = 1245 infants). In further analyses, a set of mothers who had been pregnant before and after weight loss surgery were found to have gained 50% less weight during their postsurgery pregnancy. The frequency of macrosomia was 88% lower in the postsurgery offspring and the proportion of children who were severely obese decreased by 70% [47]. These children were also noted to have lower blood glucose, insulin, and triglyceride levels. After surgery, mothers had significant reductions in BMI, blood glucose, insulin, lipid, and adipokine levels. These maternal metabolic changes likely reflect changes in the in utero environment for the fetus, suggesting that the differences in the metabolic environment aftersurgery influenced child weight outcomes. While the authors could not control for other environmental exposures and the influence of genetics, studies in animals where the mother's metabolic profile has been altered while controlling for genetic and environmental influences found similar changes in the offspring's weight as seen in this human study [34].

Several studies have also shown that excessive gestational weight gain and its associated alterations in the intrauterine environment can contribute not only to overly rapid fetal and infant growth but to the future risk of childhood overweight [48, 49]. In the Growing Up Today Study cohort, Oken and colleagues [50] were able to confirm that greater weight gain during pregnancy, controlling for maternal prepregnancy BMI, was associated with increased risk of obesity in adolescents (OR = 1.42, 95% CI 1.19–1.70). There were also greater odds of having a large-for-gestational-age (LGA) infant if the mothers gained 45 lbs or more compared to those mothers who gained 20–24 lbs (OR = 4.14, 95% CI 3.33–5.15). This study was unable to tease apart the effect of shared maternal-child dietary environments and shared genetics. However, controlling for maternal BMI helped to demonstrate that excessive weight gain itself can also affect child weight status. Other studies have shown that excess weight gain and hyperglycemia in mothers can result in fetal hyperinsulinemia, which has been associated with high birth weight and impaired glucose tolerance in adolescence [51].

In addition to weight gain, recent research has found that maternal dietary intake (specifically low carbohydrate intake) during early pregnancy was associated with gene methylation linked with higher offspring weight [52]. Other studies have also found independent associations between maternal dietary intake during pregnancy and offspring weight [53], adolescent blood pressure [54], and dietary patterns [55]. These data, in conjunction with animal data showing changes in hypothalamic gene expression when rats are exposed to certain foods in utero [36, 37], suggest that epigenetic changes may be partially mediating the effects of excess gestational weight gain and increased obesity risk in children.

In addition to gestational weight gain, gestational diabetes (GDM), which is marked by new onset hyperglycemia and insulinemia during pregnancy, has been considered another phenomenon that has the potential to alter the in utero environment and subsequent phenotypic expression of genes. In a cohort of large-for-gestational-age (LGA) and appropriate-for-gestational-age (AGA) children born to mothers with and without GDM, children who were LGA and born to mothers with GDM had the greatest risk of developing metabolic syndrome [56]. Another study showed that children born to mothers with GDM had increased odds of being overweight in adolescence (controlling for maternal SES, breast feeding history, smoking, and child diet and physical activity) [57]. However, this relationship was attenuated after taking into account birth weight and maternal weight. This study suggests that the metabolic changes that occur to the in utero environment when the mother develops gestational diabetes affects the child's birth weight, which then influences the child's risk for developing obesity. However, the fact that maternal weight attenuated the relationship calls into question whether there are other genetic factors that are passed on from mother to child that predispose both mother and child to increased weight and/or diabetes-like characteristics. These common genetic factors may ultimately make them more at risk when exposed to toxic nutritional environments postnatally.

 Outside of maternal weight gain and metabolic disorders, maternal smoking is an environmental factor that seems to affect child weight status. An observational study in Australia using a cohort of 3253 children demonstrated that mothers who smoked during pregnancy compared to nonsmokers had higher odds of having overweight (OR = 1.30, 95% CI 1.05–1.60) or obese (OR = 1.40, 95% CI 1.01–1.94) children at 14 years of age, controlling for maternal education, income, breast feeding history, and child's diet and physical activity [58]. Their study suggests that smoking specifically during the gestational period may have an impact on the development of the child. Infants born of smoking mothers appear to be smaller for gestational age or have lower birth weight than those of nonsmoking mothers [59–61], yet have a greater percentage of body fat and lower percentage of lean mass [62]. A recent study, however, found that providing folic acid supplements to mothers who smoked reduced their risk of having children with low birth weight [63]. Folic acid is a major methyl donor known to epigenetically alter the expression of genes. Thus, while smoking appears to impact one's risk for obesity, other nutritional factors like folic acid may be able to counteract these effects, demonstrating the plasticity of the human genome during the prenatal period.

Finally, greater weight gain velocity during the first year of life is associated with up to a fivefold increased risk of obesity later in life [64–66]. While the relationship between absolute birth weight and later risk for obesity is less clear [67], the relationship between accelerated catch-up growth and obesity risk is particularly salient among children who are born small for gestational age [68]. In industrialized nations, children who gain weight quickly during the first year of life seem to be particularly prone to later obesity, insulin resistance, and cardiovascular disease [69–71]. These findings have caused many providers to question the optimal rate of catch-up growth. However, the same genetic factors that make one child more likely to have excess catch-up growth may also affect their risk for obesity. Thus early catch-up growth may simply be a manifestation of one's genetic potential to store excess energy as fat and become overweight.

## 6. Discussion

 While we are unable to alter which genes we are born with, findings from animal and human studies suggest that we may be able to affect the expression of these genes and therefore our risk for future disease states. It is still unclear whether or not interventions can help curb the genetic influence of obesity. However, there are several potential lines for future investigation.

One area worth exploring is how to reduce excess gestational weight gain. It appears that excess weight gain in pregnancy has deleterious effects on child weight and increases risk for delivering heavy infants [72–74]. However, it is still unclear how best to limit excess gestational weight gain and what the impact of such interventions is on offspring growth and development. In 2009, the IOM updated their recommendations to limit weight gain during pregnancy to 15–25 lbs. for overweight women and 11–20 lbs. for obese women [75]. However, reports suggest that 50% to 60% of overweight women gain more than recommended [74, 76].

At this time, few studies have evaluated interventions during pregnancy to promote weight gain within these recommendations [77]. Outside of bariatric surgery, weight control studies for pregnant women suggest that lifestyle changes that include behavioral counseling, monitoring weight gain, limiting caloric intake, and increasing physical activity may curb excessive gestational weight gain [78]. In one study of women with gestational diabetes, dietary and physical activity modifications were related to lower infant birth weight and lower risk for-large-for-gestational-age newborns, despite minimal changes in GDM risk [79]. Unfortunately, most research to date has not been randomized and contains sample sizes too small to examine the impact of interventions on offspring weight trajectories. Nevertheless, available research suggests that limiting gestational weight gain has not been associated with increased adverse outcomes for the infant in the neonatal period [72–74]. Ongoing research by the LIFE-MOMS NIH consortium of studies will help inform whether reducing gestational weight gain in obese women can reduce the risk of offspring obesity. More work is needed in order to understand how best to limit excess gestational weight gain or GDM in women, increase the effectiveness of lifestyle interventions, and understand how these interventions influence offspring obesity risk.

 Maternal nutritional exposure and dietary composition during pregnancy is another area that requires attention. As seen in animal studies, exposure to certain methylating agents and the quality/quantity of maternal dietary intake during pregnancy can influence the expression of certain hormones and neuropeptides in the infant and the subsequent development of obesity. Similar methyl donor studies in humans would be difficult to conduct because of ethical considerations and the difficulty in controlling for the myriad of other influences that affect a child's risk for obesity. However, randomized trials could test the influence of maternal dietary prescriptions during pregnancy (e.g., low glycemic diet, low-fat diet, low-energy-dense diet) on maternal/fetal outcomes. Such research should include repeat assessments of maternal diet during pregnancy and infant intake postpartum. At the same time, additional animal studies will need to be conducted to more precisely determine the effect of specific nutritional elements and diets on hormone levels, gene expression, and obesity risk in the offspring, as well as determine the timing of these exposures during pregnancy.

With regards to postnatal influences, breastfeeding has been associated with a decreased risk of obesity in children [80]. However, it is unclear whether children born to overweight mothers or mothers with excessive weight gain during pregnancy can reduce their child's risk of obesity through breastfeeding. While some observational studies suggest that breastfeeding in this situation may be protective for future child overweight and the development of type 2 diabetes [81], other studies do not [82]. Unfortunately, a randomized control trial around breastfeeding similar to the animal studies that involve cross-fostering would be difficult to propose in humans. Therefore, prospective studies that follow the growth trajectory of children over time and take into account the overweight mother's prepregnancy weight, gestational weight gain, metabolic parameters, detailed feeding history during the first year of life as well as the child's subsequent diet and physical activity history may help to better clarify the impact of breast feeding on human risk for obesity among those born to mothers who are overweight, had excess gestational weight gain or diabetes during pregnancy. Given the animal studies on HC milk, a comparison between formula and breast milk consumption in infants born to overweight mothers would also be of interest.

While we know that epigenetic changes can occur in the postnatal period, it is uncertain how long after birth changes in hormone and neuropeptide expression can be altered. To date, few studies have been conducted that address the impact of feeding behaviors and nutritional quality (outside of breast feeding) in infancy and early childhood. Currently, several studies are being conducted (in the United States and Australia) that are exploring the efficacy of maternal education around nutrition and feeding behaviors during infancy on later child dietary habits, physical activity, sedentary activity, and weight gain [83–85]. While the results of these studies are pending, another study conducted in Turku, Finland (the STRIP Baby project) intervened on fat intake among infants starting at 7 months of age. They showed that children in the intervention group had decreased cholesterol and saturated fat consumption [86], as well as lower serum cholesterol levels, LDL levels, enhanced endothelial function, and lower blood pressure as they entered adolescence [87–89]. Unfortunately, their study did not focus on overall calorie restriction and its impact on obesity status was only significant among girls [90]. We also do not know whether these dietary changes had an effect on gene expression. Nevertheless, more studies like these are needed to determine the quality and quantity of nutritional intake in the postnatal period as well as the intensity of the interventions that are needed to curb the risk for obesity. Similar studies in SGA and LGA infants would be particularly useful since these children manifest a greater risk for obesity in later life and their optimal rate of growth in the neonatal period is still in question. However, animal studies will still be needed in order to examine the metabolic and molecular impact of these interventions and determine how long after birth epigenetic changes can occur.

The ultimate goal will be to develop systems-wide environmental or policy changes (like the recent IOM recommendations for gestational weight gain) that take advantage of the mounting epigenetic evidence around obesity risk. At this time, most of the studies in this area are laboratory-based animal studies where exposures can be controlled and testing for epigenetic evidence of change is more feasible. Translating the results of these studies to humans may involve designing community-based intervention studies comparing the effects of different approaches (e.g., nutritional interventions in healthcare settings) on offspring obesity risk. Other nonrandomized studies could involve comparisons among clearly defined closed cohorts where, for example, access to certain nutritional additives is currently limited, religious or cultural practices prescribe limits on the intake of certain foods (like animal fat or protein) or during natural events that result in nutritional deprivation. Because of the inability to control for all influences and examine biological or molecular mechanisms on a populationwide level, we will frequently have to rely on animal studies to clarify the possible mechanisms of action. As greater evidence develops, policy wide changes regarding nutritional additives to food products (like Vitamin D in milk and iron in cereals), dietary recommendations during pregnancy, the composition of formula, the optimal timing of the introduction of solids, and the optimal rate of growth may help to decrease the risk for obesity in certain populations.

Genes can greatly affect one's risk of obesity. However, there are several epigenetic influences that can alter the expression of our genes and ultimately our risk of becoming obese. These factors, in conjunction with the obesogenic environment, result in a complex web of influence that can be difficult to change. At this point, we are beginning to understand the importance of maternal gestational weight and nutritional factors on offspring obesity risk. We also know that epigenetic changes can occur prenatally, postnatally and sometimes can be carried over to the next generation without any additional manipulations. The overarching influence of these epigenetic changes help to explain the growing prevalence of obesity around the world in a time when genomic changes have not occurred. However, more work needs to be done to determine if there are critical periods for these effects, the impact of other nutritional elements and dietary compositions during pregnancy and the postnatal period, and the impact of other environmental toxins on epigenetic expression. Given the limitations of human studies, laboratory-based animal studies will provide insight regarding the underlying mechanisms of action. Once mechanisms and timing of action are determined, additional studies will be needed to develop novel interventions that effect behavior change and, ultimately, study the impact of policy changes on obesity rates. Because of the developments in the field of epigenetics, we now have new insight into the plausible mechanisms that explain the rise in obesity worldwide. With this information, we will be able to develop novel interventions for potential critical periods (i.e., pregnancy and early childhood) to reduce the lifetime risk of obesity.
